# One‐stage fluoroscopy‐guided laparoscopic transcystic papillary balloon dilation and laparoscopic cholecystectomy in patients with cholecystocholedocholithiasis who previously had undergone gastrectomy for gastric cancer

**DOI:** 10.1111/ases.12845

**Published:** 2020-08-12

**Authors:** Teppei Kamada, Hironori Ohdaira, Hideyuki Takeuchi, Junji Takahashi, Rui Marukuchi, Eisaku Ito, Norihiko Suzuki, Satoshi Narihiro, Sojun Hoshimoto, Masashi Yoshida, Eigoro Yamanouchi, Yutaka Suzuki

**Affiliations:** ^1^ Department of Surgery International University of Health and Welfare Hospital Nasushiobara Japan; ^2^ Department of Radiology International University of Health and Welfare Hospital Nasushiobara Japan

**Keywords:** cholecystocholedocholithiasis, gastrectomy, transcystic papillary balloon dilation

## Abstract

**Background:**

Patients with a history of gastrectomy have a higher incidence of cholecystocholedocholithiasis (CCL) and related morbidities than the general population. However, the management of common bile duct (CBD) stones with endoscopic retrograde cholangiopancreatography is challenging in patients after Roux‐en‐Y or Billroth II reconstruction because of the altered gastrointestinal anatomy. The aim of the current study was to evaluate the safety and efficacy of one‐stage laparoscopic transcystic papillary balloon dilation and laparoscopic cholecystectomy (LTPBD+LC) in patients with previous gastrectomy for gastric cancer.

**Methods:**

This retrospective cohort study included five patients with CCL who had previously undergone gastrectomy. All five underwent LTPBD+LC between May 2015 and February 2020 at our institution. The primary end‐point was complete clearance of the CBD stones.

**Results:**

Of the 311 patients who had undergone gastrectomy for gastric cancer from December 2009 to December 2018 at our institution, six (1.9%) were later diagnosed with CCL. Five of the six patients did not need emergency biliary drainage and underwent conservative therapy and subsequent elective LTPBD+LC. LTPBD+LC was successfully performed in all cases. None of the patients required conversion to open surgery. The rate of complete clearance of the CBD stones was 100%. The mean operative time of the entire procedure was 126 minutes (range, 102‐144 minutes), and the mean blood loss was 12.4 mL (range, 1‐50 mL). There were no major perioperative complications, and the mean length of postoperative hospital stay was 4.2 days (range, 3‐7 days).

**Conclusion:**

One‐stage LTPBD+LC may be a feasible procedure for patients with CCL who have previously undergone gastrectomy for gastric cancer.

## 1 INTRODUCTION

The risk of cholecystocholedocholithiasis (CCL) after gastrectomy for gastric cancer has been reported in many studies.^1^, ^2^, ^3^, ^4^ In patients who have undergone gastrectomy for gastric cancer, treatment options for CCL include two‐stage endoscopic retrograde cholangiopancreatography (ERCP) followed by laparoscopic cholecystectomy (LC), one‐stage laparoscopic common bile duct exploration (LCBDE) and LC (LCBDE+LC), and open common bile duct exploration and open cholecystectomy.^5^, ^6^, ^7^ However, the presence of a gastrojejunal anastomosis after gastrectomy, such as with Billroth II (B‐II) or Roux‐en‐Y (R‐Y) reconstruction, has traditionally presented a challenge in subsequent ERCP, which has a low success rate.^5^, ^8^

In cases of failed ERCP, LCBDE+LC is usually performed. Typically, LCBDE is performed via a transductal approach, or a transcystic extraction of common bile duct (CBD) stones is performed using choledochoscopy or a T‐tube. However, these procedures may be associated with several problems, including bile duct stricture, bile leakage, and limitations due to the size of the stones.^9^, ^10^

Since 2015, we have treated patients with CCL after gastrectomy with one‐stage laparoscopic transcystic papillary balloon dilation (LTPBD), pressure washing, and LC (LTPBD +LC). The aim of the current study was to evaluate the safety and efficacy of LTPBD+LC for patients with previous gastrectomy for gastric cancer.

## 2 MATERIALS AND METHODS

Of the 311 patients who had previously undergone gastrectomy for gastric cancer at our institution from December 2009 to December 2018, six patients (1.9%) were subsequently diagnosed with CCL. The diagnosis of CCL was based on the findings of enhanced CT and blood tests that were performed as part of the regular follow‐up for gastric cancer.

In one of the six patients, ERCP was unsuccessful, and the patient required emergency biliary drainage; this patient was successfully treated with lithotomy by percutaneous transhepatic gallbladder drainage and elective LC. The remaining five patients did not need emergency biliary drainage and underwent conservative therapy followed by elective LTPBD+LC. The five patients who underwent LTPBD+LC between May 2015 and February 2020 were retrospectively enrolled and evaluated in this study.

In each of the five patients, the number and size of the CBD stones were determined by magnetic resonance cholangiopancreatography (MRCP). The five operations were performed by four surgeons (three senior residents and one hepato‐biliary‐pancreatic surgeon). For this study, patients' demographic data (age, sex, BMI, ASA Physical Status [ASA‐PS] score, and comorbidities), surgical procedure data (intraoperative complications, operative time, blood loss, and open conversion), and outcome data (blood tests, postoperative complications, recurrence rate, and length of postoperative hospital stay) were collected from their hospital medical records.

The patients were followed up every 3 months for gastric cancer and CCL by blood tests (every 3 months) and enhanced CT (every 6 months). The primary end‐point was complete clearance of the CBD stones, and the secondary end‐points were recurrence rate, postoperative complications, and length of postoperative hospital stay.

This study was approved by the institutional review board of the International University of Health and Welfare Hospital (Nasushiobara, Japan). The study was performed in accordance with the Declaration of Helsinki, and informed consent for study participation was obtained from the patients.

### 2.1 Surgical procedure

The procedure was performed under constant fluoroscopic guidance by a team of surgeons and an interventional radiologist. A typical four‐trocar technique with two 12‐mm and two 5‐mm trocars was used for LC.

**FIGURE 1:**
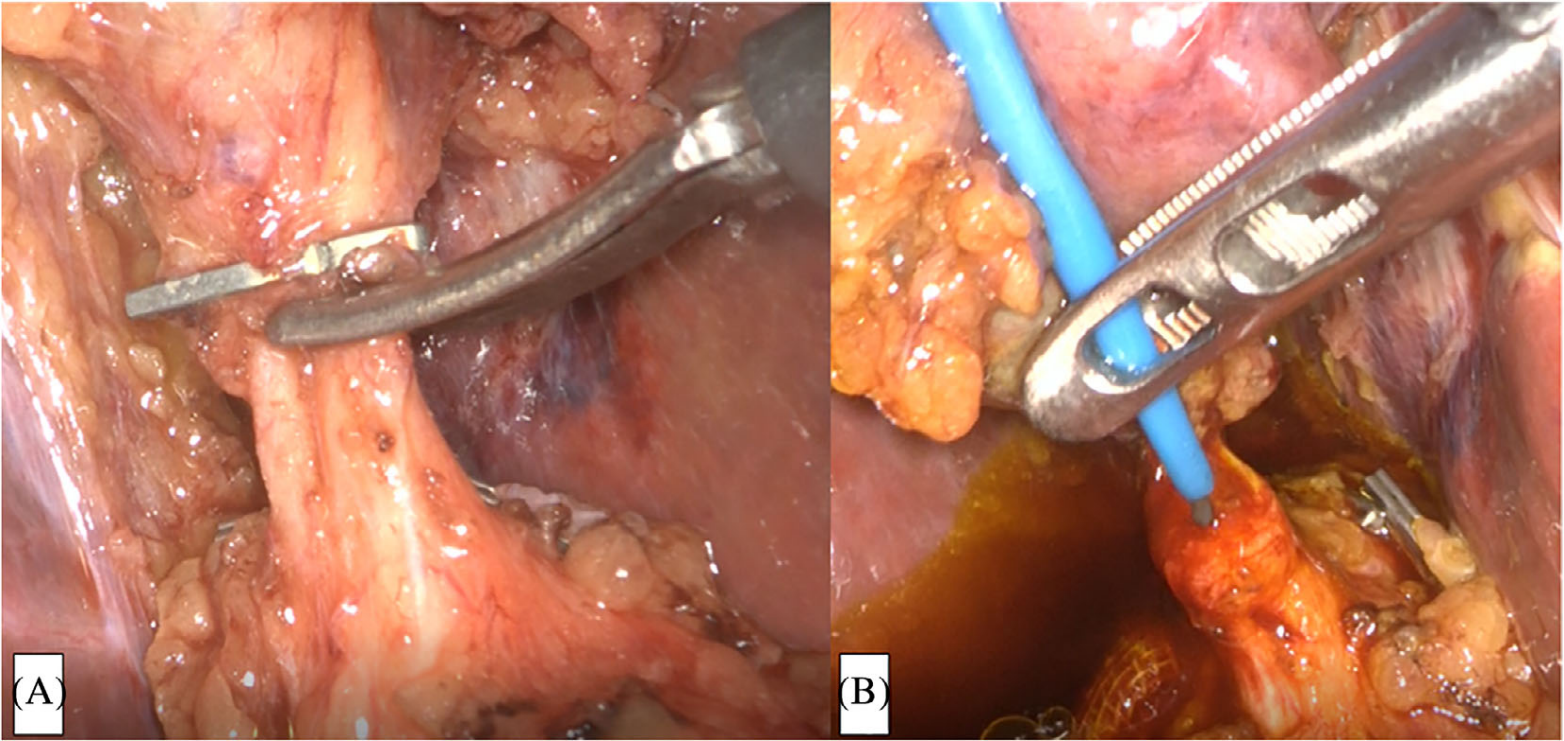
Intraoperative imaging of one‐stage laparoscopic transcystic papillary balloon dilation and laparoscopic cholecystectomy. A, The common bile duct is cut halfway around the circumference. B, A BRITE TIP sheath introducer is inserted into the cystic duct

First, standard LC was performed while ensuring a critical view of safety. Specifically, dissection aimed to expose and delineate the hepatocystic triangle completely, enable identification of a single duct and a single artery entering the gallbladder, and completely dissect the lower part of the gallbladder off the liver bed.^11^, ^12^ The cystic artery was clipped and transected. After the cystic duct was clipped on the gallbladder side and cut halfway around its circumference (Figure 1A), a BRITE TIP Interventional Sheath Introducer (Cordis, Warren, New Jersey) was percutaneously inserted into the cystic duct via the right hypochondrial region with a flexible Radifocus Guidewire (Terumo Europe NV, Leuven, Belgium) (Figure 1B). The guidewire was cannulated into the duodenum beyond the papilla of Vater through the CBD.

**FIGURE 2:**
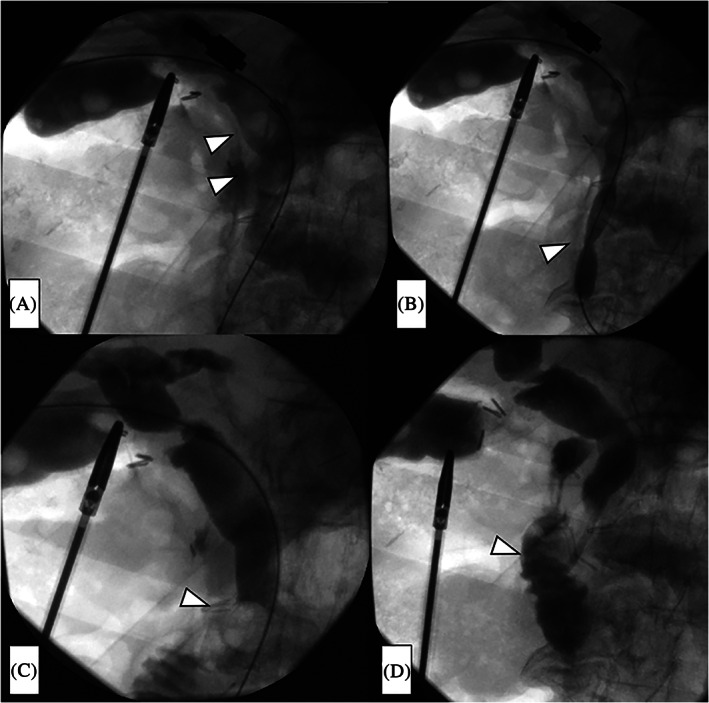
Radiographic images during one‐stage laparoscopic transcystic papillary balloon dilation and laparoscopic cholecystectomy. A, The presence of choledocholithiasis was confirmed by injecting contrast medium into the bile duct from the sheath (arrowheads). B, Balloon dilation of the papilla of Vater (arrowhead). C, The stones were extruded using the balloon (arrowhead), and the common bile duct was cleaned. D, The absence of any residual stones was confirmed, and a pigtail‐shaped drainage catheter (arrowhead) was inserted to help prevent edema of the papilla of Vater and pancreatitis

After contrast medium was injected into the bile duct from the sheath and the presence of choledocholithiasis was confirmed (Figure 2A), the papilla of Vater was dilated using a TMP balloon catheter (10 × 40 mm; 7 atm, 3 minutes) (Tokai Medical Products, Aichi, Japan) (Figure 2B).

A Selecon MP Catheter (Terumo Europe NV) was then inflated upstream of the choledocholithiasis, after which the stones were extruded using the catheter balloon and the CBD was cleaned (Figure 2C). This procedure was repeated three times.

After the CBD was cleaned, the absence of residual stones was confirmed by a final cholangiogram, and a pigtail‐shaped drainage catheter (UreSil, Skokie, Illinois) was placed at the papilla of Vater to prevent edema of the papilla of Vater and subsequent pancreatitis (Figure 2D). This drainage catheter easily falls into the duodenum when oral feeding is commenced. Finally, we removed the balloon catheter system, closed the cystic duct with clips, and extracted the gallbladder in the usual manner.

**FIGURE 3:**
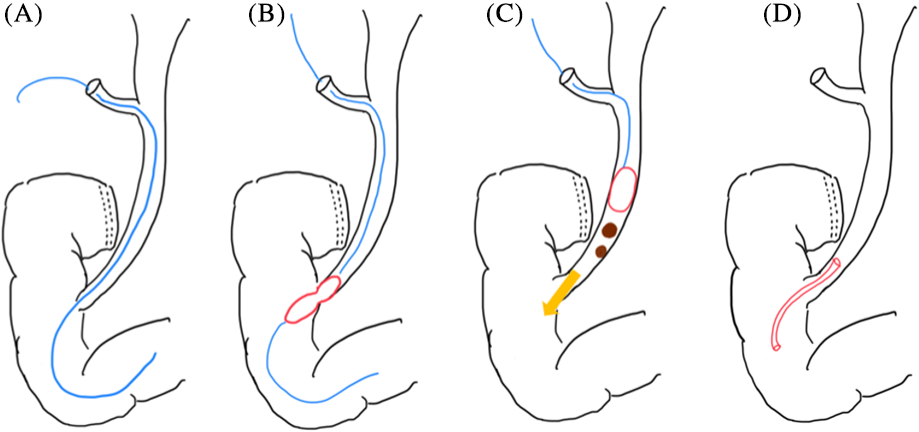
Schema of one‐stage laparoscopic transcystic papillary balloon dilation and laparoscopic cholecystectomy. A, The guidewire was cannulated into the duodenum beyond the papilla of Vater through the common bile duct. B, Balloon dilation of the papilla of Vater. C, The stones were extruded with the balloon, and the common bile duct was cleaned. D, Placement of a pigtail‐shaped drainage catheter

The schema of LTPBD+LC is presented in Figure 3A‐D.

## 3 RESULTS

All five patients were men, with a mean age of 77 years (range, 65‐92 years). All of them had an ASA‐PS score of 3 (Table 1). The mean preoperative BMI was 19.3 kg/m^2^ (range: 15.4‐24.3).

**TABLE 1:** Characteristics of the five patients who underwent one‐stage laparoscopic transcystic papillary balloon dilation and laparoscopic cholecystectomy

Case	Age (y), sex	BMI (kg/m^2^)	Pathological stage	Gastrectomy reconstruction	Number of stones	Maximum Diameter of stones (mm)	Total operative time; fluoroscopy time (min)	Severity of adhesions around HDL	Length of hospital stay (d)	Blood loss (mL)	Follow‐up period (mo)	Complications
1	73, M	20.2	IA	LTG R‐Y	1	2	102; 29	Mild	3	3	15	None
2	92, M	20.8	IB	LDG R‐Y	Multiple	12	133; 28	Severe	3	1	3	None
3	65, M	24.3	IA	LDG B‐II	1	6	144; 26	Severe	4	5	0	None
4	65, M	15.4	IIIB	LTG R‐Y	1	5	111; 40	Mild	4	50	51	None
5	90, M	15.9	IIA	LDG R‐Y	Multiple	12	142; 42	Moderate	7	3	54	None

Abbreviations: B‐I, Billroth‐ I; B‐II, Billroth‐ II; HDL, hepatoduodenal ligament; LDG, laparoscopic distal gastrectomy; LTG, laparoscopic total gastrectomy; M, male; R‐Y, Roux‐en‐Y.

In each case, MRCP was able to preoperatively detect the presence of CBD stones, as well as their size and number.

Before LTPBD+LC, three patients had undergone laparoscopic distal gastrectomy previously, and two had undergone laparoscopic total gastrectomy. Two patients had received postoperative adjuvant chemotherapy with tegafur/gimeracil/oteracil. Four patients had undergone R‐Y reconstruction, and one had undergone B‐II reconstruction. Three patients had undergone D1+ lymph node dissection, and two had undergone D2 lymph node dissection.

Pathological diagnosis of the gastric cancer showed that three patients had stage I disease, one had stage II, and one had stage III. Lymph node dissection and pathological diagnosis were performed according to the gastric cancer treatment guidelines of the Japanese Gastric Cancer Association.^13^

The mean period from gastrectomy to LTPBD+LC was 60 months (range: 3‐111 months). LTPBD+LC was successfully performed in all five cases. However, two patients had severe adhesions around the hepatoduodenal ligament, two had mild adhesions, and one had moderate adhesions. None of the patients required conversion to open surgery. The rate of complete clearance of the CBD stones was 100%. The mean operative time for the entire procedure (LTPBD+LC) was 126 minutes (range, 102‐144 minutes), and mean fluoroscopy time was 33 minutes (range, 26‐42 minutes). The mean blood loss was 12.4 mL (range, 1‐50 mL).

On postoperative day 1, blood tests in two patients showed evidence of an increase in amylase, which then normalized during the next 48 hours. None of the patients showed any clinical signs of pancreatitis, such as epigastric pain. There were no major perioperative complications, and all patients were discharged by postoperative day 7 (mean length of postoperative hospital stay, 4.2 days; range, 3‐7 days). None of the patients developed symptomatic choledocholithiasis from retained stones during the follow‐up period (mean, 24.6 months; range, 1‐54 months).

## 4 DISCUSSION

Patients with a history of gastrectomy have a higher incidence of gallstones and morbidities requiring surgical treatment than the general population. In a study comparing patients who had undergone gastrectomy with matched controls, gastrectomy was found to increase the risk of gallstones (adjusted hazard ratio = 1.77, 95% confidence interval = 1.34‐2.35).^14^ Approximately 10% of patients with gallstones have concomitant CBD stones (CCL),^15^, ^16^ which are related to serious complications, such as cholangitis and pancreatitis.

According to the literature, the causes for the increased risk of CCL include resection of the hepatic branch of the vagus nerve, non‐physiological reconstruction of the gastrointestinal tract, infection of the biliary tract, and altered response to and secretion of cholecystokinin.^17^, ^18^ However, only a few studies have examined treatment strategies for patients with CCL and a history of gastrectomy.

Zhang et al indicated that ERCP followed by LC seems to be an attractive option for treating CCL in patients with a history of B‐I gastrectomy, but LCBDE+LC appears better suited to patients with a history of B‐II or R‐Y esophagojejunostomy.^5^ In their study, the success rate of ERCP for CBD stone clearance was 81.2% in patients with a history of B‐I gastrectomy, but only 23.7% in patients with a history of non‐B‐I gastrectomy. However, the success rate of LCBDE+LC after ERCP failure was 87.7% in patients with preoperative intra‐abdominal adhesion evaluation scores ≤3 points.^5^

Kim et al reported the advantages of a one‐stage approach to the management of CBD stones after gastrectomy and noted that LCBDE+LC should be the initial approach for gastrectomy patients with CBD stones.^8^ In their study, to clear CBD stones, ERCP was attempted before LC in eight patients with previous gastrectomy, but it was successful in only one patient (12.5%). Conversely, duct clearance was successful in all patients who underwent LCBDE or open common bile duct exploration.^8^

These studies confirmed that LCBDE+LC using a transductal approach is safe and feasible for the management of CBD stones after gastrectomy. However, conventional LCBDE (ie, a transductal approach using a T‐tube, choledochoscopy, or primary closure) is associated with various complications, including bile duct stricture, bile leakage, prolonged biliary fistula, bleeding, and possible peritonitis after T‐tube removal.^9^, ^10^

According to a systematic review and meta‐analysis of 2938 patients, the overall perioperative complication rate of LCBDE+LC by the transductal approach was 13.7%, and the biliary complication rate was 7.0%.^9^ In addition, history of gastrectomy represents a risk factor for severe intra‐abdominal adhesions around the hepatoduodenal ligament. Severe adhesions around the hepatoduodenal ligament can present challenges in performing LCBDE+LC via a transductal approach.^19^ Another method of conventional LCBDE is the transcystic approach using a grasper or basket catheter.^20^ However, according to the Society of American Gastrointestinal and Endoscopic Surgeons guidelines, transcystic stone clearance may be hampered by the presence of large (>6 mm) or numerous (>5) stones.^21^, ^22^, ^23^, ^24^

Our LTPBD+LC method has several interesting features. One of its principal advantages is the preservation of the CBD and papillary function. LTPBD+LC does not require an incision in the CBD or papilla of Vater or an insertion of a T‐tube; therefore, it is likely to reduce the risk of biliary complications (bile leakage, biliary stricture). In addition, retrograde cholangitis, which is a major complication of endoscopic sphincterotomy, does not occur with our method.

Another advantage of our method is the low invasiveness and short hospital stay required. Given this advantage, our approach might be feasible for elderly individuals with complicated comorbidities, resulting in ASA‐PS scores of 3 or 4.

The most remarkable advantage of our LTPBD+LC method is that it is a simple procedure that does not require dissection around the hepatoduodenal ligament. Thus, it does not require the level of surgical experience needed for conventional LCBDE by the transductal approach, making it possible for LTPBD+LC to be performed by resident doctors.

Although large stones (>1 cm) and multiple stones (>5) were present in some of our patients, complete CBD clearance was obtained by LTPBD+LC. This suggests that the decision to perform LTPBD+LC should not be based only on the size and number of CBD stones.

Additionally, previous authors have reported the feasibility of one‐stage procedures using the same concept as our LTPBD+LC for CCL patients,^25^, ^26^ and our results corroborate those of these previous studies. Given the advantages of LTPBD+LC, the procedure seems most suitable for patients with CCL who could not be treated by ERCP or who have severe ASA‐PS scores, such as patients with previous gastrectomy. Also, surgical outcomes of our procedure in patients with CCL who had undergone previous gastrectomy were similar to those of previous LTPBD studies.^25^, ^26^

Our method of LTPBD+LC did have some disadvantages. Some systematic reviews generally suggest that endoscopic papillary balloon dilation is inferior to endoscopic sphincterotomy in terms of overall stone removal and increased risk of pancreatitis.^27^, ^28^

Although our study showed an acceptable rate of complete clearance of CBD stones, it included only a limited number of cases. As such, the results cannot be generalized to stones of different sizes and in all locations. For example, in cases of CBD stones located in the intrahepatic bile duct or larger than 15 mm, LTPBD+LC might not be effective. In such cases, LCBDE via a transductal approach or intraoperative ERCP would be necessary. Furthermore, there may be an increased risk of pancreatitis after LTPBD+LC if the pancreatic duct becomes compressed due to edema of the papilla of Vater. Prevention of pancreatitis after LTPBD+LC is very important, and to minimize edema of the papilla of Vater, we placed a pigtail‐shaped drainage catheter. As a result, we did not encounter symptomatic pancreatitis in any of our cases.

This study had several limitations. It was a single‐institution retrospective study with a limited number of cases. Therefore, future long‐term follow‐up will be necessary to determine whether LTPBD+LC is a safe and feasible procedure for patients with previous gastrectomy. Also, LTPBD+LC was performed only as an elective procedure in cases with few intra‐abdominal adhesions. As such, it is unknown whether LTPBD+LC is feasible for acute cholecystitis or cholangitis that needs emergency biliary drainage. However, this procedure is probably contraindicated in patients with obstruction of the cystic duct due to severe inflammation or abnormal anatomy of the cystic duct that cannot be identified by preoperative MRCP.

Although one‐stage LTPBD+LC might be a useful alternative to a two‐stage approach or conventional LCBDE+LC in patients with CCL who have undergone previous gastrectomy, careful case selection is necessary.

## CONFLICTS OF INTEREST

The authors have no conflicts of interest to declare and received no financial or material support for this study.

## AUTHORS' CONTRIBUTIONS

Study design, data analysis, and writing: T.K.

Critical revision of the manuscript: E.I., H.O., and E.Y.

Figure creation: H.T.

Data collection: T.K., J.T., R.M., N.S., S.N., S.H., and M.Y.

Final approval of the manuscript: Y.S.

All authors read and approved the final manuscript.

## Data Availability

The data that support the findings of this study are available from the corresponding author upon reasonable request.

## References

[1] Liang TJ , Liu SI , Chen YC , et al. Analysis of gallstone disease after gastric cancer surgery. Gastric Cancer. 2017;20(5):895‐903. 10.1007/s10120-017-0698-5. 28154944

[2] Gillen S , Michalski CW , Schuster T , Feith M , Friess H , Kleeff J . Simultaneous/incidental cholecystectomy during gastric/esophageal resection: systematic analysis of risks and benefits. World J Surg. 2010;34(5):1008‐1014. 20135313 10.1007/s00268-010-0444-1

[3] Akatsu T , Yoshida M , Kubota T , et al. Gallstone disease after extended (D2) lymph node dissection for gastric cancer. World J Surg. 2005;29(2):182‐186. 15654665 10.1007/s00268-004-7482-5

[4] Hauters P , de Neve de Roden A , Pourbaix A , Aupaix F , Coumans P , Therasse G . Cholelithiasis: a serious complication after total gastrectomy. Br J Surg. 1988;75(9):899‐900. 3179669 10.1002/bjs.1800750923

[5] Zhang M , Zhang J , Xu Sun XJ , Zhu J , Yuan W , Yan Q . Clinical analysis of treatment strategies to cholecystocholedocholithiasis patients with previous subtotal or total gastrectomy: a retrospective cohort study. BMC Surg. 2018;18(1):54. 10.1186/s12893-018-0388-1. 30092786 PMC6085697

[6] Vannijvel M , Lesurtel M , Bouckaert W , et al. A survey of European‐African surgeons' management of common bile duct stones. HPB (Oxford). 2016;18(12):959‐964. 27838253 10.1016/j.hpb.2016.10.007 PMC5144544

[7] Quaresima S , Balla A , Guerrieri M , Campagnacci R , Lezoche E , Paganini AM . A 23 year experience with laparoscopic common bile duct exploration. HPB (Oxford). 2017;19(1):29‐35. 27890483 10.1016/j.hpb.2016.10.011

[8] Kim J , Cho JN , Joo SH , Kim BS , Lee SM . Multivariable analysis of cholecystectomy after gastrectomy: laparoscopy is a feasible initial approach even in the presence of common bile duct stones or acute cholecystitis. World J Surg. 2012;36(3):638‐644. 22270995 10.1007/s00268-012-1429-z

[9] Hajibandeh S , Hajibandeh S , Sarma DR , et al. Laparoscopic transcystic versus transductal common bile duct exploration: a systematic review and meta‐analysis. World J Surg. 2019;43(8):1935‐1948. 10.1007/s00268-019-05005-y. 30993390

[10] El‐Geidie AA . Is the use of T‐tube necessary after laparoscopic choledochotomy? J Gastrointest Surg. 2010;14(5):844‐848. 20232173 10.1007/s11605-009-1133-y

[11] Yegiyants S , Collins JC . Operative strategy can reduce the incidence of major bile duct injury in laparoscopic cholecystectomy. Am Surg. 2008;74(10):985‐987. 18942628

[12] Avgerinos C , Kelgiorgi D , Touloumis Z , Baltatzi L , Dervenis C . One thousand laparoscopic cholecystectomies in a single surgical unit using the “critical view of safety” technique. J Gastrointest Surg. 2009;13(3):498‐503. 19009323 10.1007/s11605-008-0748-8

[13] Japanese Gastric Cancer Association . Japanese gastric cancer treatment guidelines 2018 (5th edition). Gastric Cancer. 2020. 10.1007/s10120-020-01042-y. PMC7790804 32060757

[14] Kim SY , Bang WJ , Lim H , Lim MS , Kim M , Choi HG . Increased risk of gallstones after gastrectomy a longitudinal follow‐up study using a national sample cohort in Korea. Medicine (Baltimore). 2019;98(22):e15932. 31145363 10.1097/MD.0000000000015932 PMC6709130

[15] Petelin JB . Laparoscopic common bile duct exploration. Surg Endosc. 2003;17(11):1705‐1715. 12958681 10.1007/s00464-002-8917-4

[16] Tazuma S . Gallstone disease: epidemiology, pathogenesis, and classification of biliary stones (common bile duct and intrahepatic). Best Pract Res Clin Gastroenterol. 2006;20(6):1075‐1083. 17127189 10.1016/j.bpg.2006.05.009

[17] Kobayashi T , Hisanaga M , Kanehiro H , Yamada Y , Ko S , Nakajima Y . Analysis of risk factors for the development of gallstones after gastrectomy. Br J Surg. 2005;92(11):1399‐1403. 16078296 10.1002/bjs.5117

[18] Yi SQ , Ohta T , Tsuchida A , et al. Surgical anatomy of innervation of the gallbladder in humans and *Suncus murinus* with special reference to morphological understanding of gallstone formation after gastrectomy. World J Gastroenterol. 2007;13(14):2066‐2071. 17465449 10.3748/wjg.v13.i14.2066 PMC4319126

[19] Zhang MJ , Cao LP , Ding GP . Successful laparoscopic common bile duct exploration in a patient with previous Billroth II gastrectomy. Int J Clin Exp Med. 2017;10(3):5480‐5485.

[20] Paganini AM , Guerrieri M , Sarnari J , et al. Thirteen years' experience with laparoscopic transcystic common bile duct exploration for stones. Effectiveness and long‐term results. Surg Endosc. 2007;21(1):34‐40. 10.1007/s00464-005-0286-3. 17111284

[21] Overby DW , Apelgren KN , Richardson W , Fanelli R . SAGES guidelines for the clinical application of laparoscopic biliary tract surgery. Surg Endosc. 2010;24(10):2368‐2386. 10.1007/s00464-010-1268-7. 20706739

[22] Tinoco R , Tinoco A , El‐Kadre L , Peres L , Sueth D . Laparoscopic common bile duct exploration. Ann Surg. 2008;247(4):674‐679. 18362631 10.1097/SLA.0b013e3181612c85

[23] Williams EJ , Green J , Beckingham I , Parks R , Martin D , Lombard M . British Society of Gastroenterology. Guidelines on the management of common bile duct stones (CBDS). Gut. 2008;57(7):1004‐1021. 18321943 10.1136/gut.2007.121657

[24] Stromberg C , Nilsson M , Leijonmarck CE . Stone clearance and risk factors for failure in laparoscopic transcystic exploration of the common bile duct. Surg Endosc. 2008;22(5):1194‐1199. 18363068 10.1007/s00464-007-9448-9

[25] Sjer AEB , Boland DM , van Rijn PJJ , Mohamad S . A decade of washing out common bile duct stones with papillary balloon dilatation as a one‐stage procedure during laparoscopic cholecystectomy. Surg Endosc. 2010;24(9):2226‐2230. 20177926 10.1007/s00464-010-0937-x PMC2939343

[26] Masoni L , Mari FS , Pietropaolo V , Onorato M , Meucci M , Brescia A . Laparoscopic treatment for unsuspected common bile duct stones by transcystic sphincter of Oddi pneumatic balloon dilation and pressure‐washing technique. World J Surg. 2013;37(6):1258‐1262. 23474857 10.1007/s00268-013-1992-y

[27] Zhao HC , He L , Zhou DC , Geng XP , Pan FM . Meta‐analysis comparison of endoscopic papillary balloon dilatation and endoscopic sphincteropapillotomy. World J Gastroenterol. 2013;19(24):3883‐3891. 23840129 10.3748/wjg.v19.i24.3883 PMC3699051

[28] Tringali A , Rota M , Rossi M , Hassan C , Adler DG , Mutignani M . A cumulative meta‐analysis of endoscopic papillary balloon dilation versus endoscopic sphincterotomy for removal of common bile duct stones. Endoscopy. 2019;51(6):548‐559. 10.1055/a-0818-3638. 30727009

